# Geo-Spatial Aspects of Acceptance of Illegal Hunting of Large Carnivores in Scandinavia

**DOI:** 10.1371/journal.pone.0068849

**Published:** 2013-07-24

**Authors:** Kristin E. Gangaas, Bjørn P. Kaltenborn, Harry P. Andreassen

**Affiliations:** 1 Hedmark University College, Faculty of Applied Ecology and Agricultural Sciences, Campus Evenstad, Koppang, Norway; 2 Norwegian Institute of Nature Research (NINA), Lillehammer, Norway; University of Kent, United Kingdom

## Abstract

Human-carnivore conflicts are complex and are influenced by: the spatial distribution of the conflict species; the organisation and intensity of management measures such as zoning; historical experience with wildlife; land use patterns; and local cultural traditions. We have used a geographically stratified sampling of social values and attitudes to provide a novel perspective to the human – wildlife conflict. We have focused on acceptance by and disagreements between residents (measured as Potential Conflict Index; PCI) towards illegal hunting of four species of large carnivores (bear, lynx, wolf, wolverine). The study is based on surveys of residents in every municipality in Sweden and Norway who were asked their opinion on illegal hunting. Our results show how certain social values are associated with acceptance of poaching, and how these values differ geographically independent of carnivore abundance. Our approach differs from traditional survey designs, which are often biased towards urban areas. Although these traditional designs intend to be representative of a region (i.e. a random sample from a country), they tend to receive relatively few respondents from rural areas that experience the majority of conflict with carnivores. Acceptance of poaching differed significantly between Norway (12.7–15.7% of respondents) and Sweden (3.3–4.1% of respondents). We found the highest acceptance of illegal hunting in rural areas with free-ranging sheep and strong hunting traditions. Disagreements between residents (as measured by PCI) were highest in areas with intermediate population density. There was no correlation between carnivore density and either acceptance of illegal hunting or PCI. A strong positive correlation between acceptance of illegal hunting and PCI showed that areas with high acceptance of illegal hunting are areas with high potential conflict between people. Our results show that spatially-stratified surveys are required to reveal the large scale patterns in social dynamics of human-wildlife conflicts.

## Introduction

Poaching or illegal killing of wildlife are part of human – wildlife conflict and contribute to the endangerment and eradication of species worldwide [1]–[8]. Some of the most prominent conflicts caused by illegal killing are related to the presence of large carnivores [3], [9], [10]. Muth and Bowe (1998) classify the motivation for poaching into 10 categories, but emphasise the motivation of local rural hunters to protect property, the traditional use of nature, and rebellion against management authorities and regulations [11], [12].

In the Scandinavian Peninsula (i.e. Norway and Sweden), populations of all four large carnivore species: brown bear (Ursus arctos), lynx (Lynx lynx), wolf (Canis lupus) and wolverine (Gulo gulo), are recovering after decades of eradication [13]. Today large carnivores occur outside protected areas, leading to conflicts with farmers and big game hunters [9]. To relieve the conflict between farmers and carnivores, wildlife management authorities have often used zoning, where they create protected areas for the focal species, as a tool to separate needs of humans and wildlife [14]. In 2003, a wolf zoning area was introduced in Norway where sheep density and domestic prey abundance were low in order to improve the protection of wolves. Despite this measure, wildlife management has found that poaching is a major cause of death of all four carnivore species, comprising 51% of the total mortality of the wolf including the zoning area [8], [9], [15]–[18]. Carnivore restoration projects all over the world experience the same challenges as in Scandinavia, as farmers are accustomed to managing domestic animals in a predator-free environment. After carnivore restoration, livestock experience an increased risk of depredation by the reintroduced or recovering predator population [19], [20]. Hence, inhabitants in rural areas generally have a more skeptical attitude towards carnivore restoration, while people living in more urban areas may express more acceptance of carnivores [21]. This rural-urban divide is traditionally interpreted as one of the core elements of the human dimension part of the conflict [14], [22]–[24].

In this paper, we take a geographical perspective by using a geographically stratified sampling of humans and mapping the level of acceptance of poaching throughout the Scandinavian Peninsula. We argue that acceptance of poaching has spatial dimensions, and that this picture may vary throughout Scandinavia, partly as a result of variation in land use and exposure to large carnivores. Attitudes toward carnivores in general and poaching in particular may have spatial dimensions, if attitudes are related to spatially variable factors such as the presence of carnivores, management practices (e.g., zoning), culture, and traditions (e.g. sheep farming and big game hunting in rural areas). In spite of the idea that zoning might relieve the conflict between sheep farmers and carnivores, it might also increase the conflict level in certain areas due to a sense of injustice in areas selected for wolf presence and loss of evenly distributed costs of having carnivores [14].

We expect that humans living in areas inhabited by carnivores will be more prone to accept poaching, as they are directly affected by carnivore presence. However, it has also been shown that people inhabiting areas where carnivores have existed for a long time tend to be more tolerant of carnivores compared to people experiencing carnivore reintroduction [25]–[27]. Hence, we have also tested if the presence of carnivores in historic time reduces the conflict.

We expected a positive association between acceptance of poaching and 1) amount of loss of sheep to carnivores; 2) degree of ruralisation including traditional use of nature (e.g. big game hunting); 3) presence of a wolf zone; and 4) recent colonization by carnivores.

We also estimated a Potential Conflict Index (PCI), where the level of consensus was measured by estimating the divergence of attitudes [28], we expected the highest conflict (PCI) in areas with large losses of sheep and anintermediate degree of ruralisation. These are rural areas where local hunters maintain old traditions and have a higher tendency to oppose urban values and rules, but still live side by side with people that have a more urban lifestyle [21], [29].

## Methods

### Survey

#### Ethics statement

Data on attitudes towards carnivores were collected through a telephone survey carried out by a data collection agency (www.norstat.no). The data collection agency (NORSTAT) bases its sample on existing registers that are publicly available when they collect data by telephone interviews. When the respondents in our study were contacted the interviewer followed a strict protocol as dictated by standard research ethics, including presenting the purpose of the study and the agency behind it, the fact that participation was entirely voluntary, how long the interview would take, and how the results would be used. The research agency commissioning the study and the data collection agency are not required to seek permission for this kind of data collection from the Norwegian Social Science Data Service (NSD). NSD is the institution reviewing research proposals and issuing permits for data collection, but an ethics review and a permit is only required in cases where the researchers and/or the data collection agency retain a register of respondents for purposes such as reminders or follow up surveys. This was not the case for our study, and we have no register or any other kind of information that can be used for linking individuals to the data set.

#### Sampling procedures

In order to obtain responses that were evenly distributed throughout Scandinavia independent of population density, we used a geographically stratified sampling by surveying 4–5 people in each municipality in Sweden and in Norway. This was important in order to assess geographical distribution of conflict and useful for comparing attitudes of people living inside and outside zoning areas, such as the wolf zone. However, as the sample represents a very small proportion of people living in high density areas such as cities and suburban areas it does not measure the general opinion of people living in a specific region (e.g. county or country).

#### Questionnaire

The questionnaire focused mainly on the respondents’ attitudes towards large carnivores in general, with some questions focusing specifically on the respondents’ attitudes toward illegal hunting (Table 1). We used Cronbach’s alpha [30] to check for internal consistency of the data [31]. Additionally we included questions on individual characteristics such as age, sex and education level. Attitudes towards illegal hunting had a Likert type response format ranging from “strongly disagree”, “disagree”, “neither agree nor disagree”, “agree”, to “strongly agree”, and was separated into each species, i.e. “Poaching of brown bear is acceptable”. It is important to note that we did not ask whether or not they were personally willing to poach, but only whether they found poaching to be acceptable.

**Table 1:** The questionnaire (16 questions in total) including the questions dealing with acceptance of illegal hunting (question 6–9).

Statements
1. Compensation should be granted only if it is implemented preventive measures
2. Any disadvantage with predators should be compensated
3. It is the responsibility of agriculture to adapt to the situation predators
4. Fear is a good enough reason to remove predators
5. There are strong traditions of hunting big game where I live
6. Poaching of brown bear is acceptable
7. Poaching of wolf is acceptable
8. Poaching of wolverine is acceptable
9. Poaching of lynx is acceptable
10. Carnivores should be managed in line with other wildlife
11. Large carnivores are an enrichment for my nature experience
12. Carnivores limit my use of nature
13. Seeing tracks and signs increase my quality of life
14. Carnivores should be utilized to a greater extent in the tourism context
15. Seeing predators in nature is a privilege
16. Norway is a rich country that should take responsibility for large predators

The questions were answered from highly disagree to highly agree on a 5 level Likert scale.

### Response Variables

In this paper we have focused on the following two response variables: 1) the acceptance of illegal hunting of all 4 large carnivore species, and 2) the conflicts between humans in association with acceptance of illegal hunting, estimated as the Potential Conflict Index (PCI).

We applied the Potential Conflict Index (PCI) to estimate the divergence of attitudes [28]. If everyone in an area agreeds that poaching was either acceptable or unacceptable the PCI would be low, while highly divergent opinions would result in high PCI values. We applied the second generation potential conflict index (PCI_2_) developed by Manfredo and Vaske [28], [32] which range from 0 to 1, where PCI_2_ = 0 indicates high consensus and therefore low conflict level, while PCI_2_ = 1 means low consensus and a potentially high conflict level [32].

PCI_2_ has been used to estimate the opinions and conflicts of hunters and their response to chronic wasting disease [33], interactions between cougars and humans in Alberta [1], what people think of restoration of archaeological ruins [34] and stakeholders attitudes to different activities offered in a certain area of Lake Umbagog in Maine [35].

The PCI_2_ estimates the distance between people who agree on a question (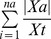), and those who disagree (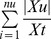): PCI = 
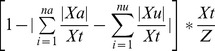
, where 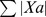 = is the sum of the n_a_ “positive” respondents; i.e. that found poaching “acceptable” (defined as 1) or “highly acceptable” (defined as 2).


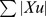
 = is the sum of the n_u_ “negative” respondents that found poaching “unacceptable” (defined as −1) or “highly unacceptable” (defined as −2).
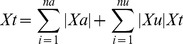



Z = is the maximum possible sum of all scores; that is n * extreme score on a scale (e.g., Z = 2n for scale going from −2 to +2) and n = n_a_+n_u_+number of neutral responses (i.e. neither nor reject illegal hunting).

### Predictor Variables

The main predictors in our analyses were: density of each of the four large carnivores today and historical time (i.e.1856–1860), presence of a wolf zone, human density and rural traditions represented by big game hunting, free grazing sheep and sheep depredated by large carnivores. In the analyses of acceptance of illegal hunting at the individual level we also included the respondent’s education level, sex and age as covariates as these has been found to be important predictors in human-wildlife conflicts [20], [36], [37]. We present significant contributions from these individual characteristics, but otherwise do not discuss them further.

Carnivore presence is based on data from national wildlife databases Norwegian “Rovbasen” (www.dirnat.no) and the Swedish “Rovdjursforum” (www.naturvardsverket.se) together with data from Statistics Norway (www.SSB.no) and Statistics Sweden (www.SCB.se). The numbers do not represent the total number of carnivores in an area, but rather the minimum number of individuals known to be present. This is done through yearly registrations of carnivore individuals based on snow-tracking and radio-tracking family groups of lynx, wolves and wolverines, counts of bear and wolverine dens, and DNA analyses of scats [38]. Historical data are based on bounties paid in the years of 1856–1860, which is the only continuous 5 year-period where we had access to registration for all four large carnivore species from both Norway and Sweden [13]. These data provide a historical window depicting how the “natural” carnivore populations were distributed before the current management interventions were introduced. A few changes have taken place in the organisation of counties since 1850, especially in Sweden where several counties have merged into fewer, but larger counties. We aggregated the numbers of each carnivore species shot per year per county into the same counties we have today.

### Statistical Procedures

#### General linear models

We used generalised linear mixed models (GLMM) to reveal associations between the responses and the various predictor variables [30]. All predictor variables were tested for multicollinearity before combined in the analyses [31].The most parsimonious models were selected by a backward elimination of non-significant terms (p>0.05) by using Likelihood ratio tests. We chose a backwards selection procedure because we were testing our hypothesis [30]. All analyses were done using statistical procedures available in R 2.13.1 (http://cran.r-project.org/).

Due to the geographically stratified sampling scheme, mean acceptance and PCI do not reflect the mean attitudes of inhabitants of a county or a country, as urban areas are underrepresented compared to a random sample from such a region.

#### Acceptance

We defined acceptance of illegal hunting as a binomial response (“agree” and “highly agree” defined as 1 vs. all other answers at the Likert scale defined as 0) and analysed acceptance with a binomial response error and logit link function. We did the main analyses on acceptance at the level of the individual respondent to be able to include our covariates on individual characteristics (i.e. sex, age and education level). In this individual based analysis we used a mixed models (GLMM) by entering municipality as a random effect. As the respondents answered according to each of the large carnivore species we included species as a predictor variable. In addition we included the main geographic predictors characterising the municipality where the respondent live: i.e. country (Norway, Sweden), density of each of the four large carnivores today and historical time (i.e.1856–1860), and human density. Wolf zone was introduced to a model with data from Norway at the municipality level as some counties are split into zones.

As the carnivore densities are at the county level and to resemble the spatial scale of the main PCI_2_ analyses, we also analysed acceptance at the county level with the mean acceptance from the Likert scale in the county as response. Because each county obtained 4 observations (one for each carnivore species) we used GLMM with normal error distribution and county as random effect.

#### Conflict

To estimate PCI_2_ we grouped individuals in a county, as it is an index describing divergence in attitudes within a group of people. We estimated how PCI_2_ correlated with illegal hunting for each of the four large carnivore species. In these analyses we used carnivore species and the geographical descriptors characterising the county of the respondent: country (Norway, Sweden), presence of each of the four large carnivores today and historically, and human density (also as a second order component) as the predictors in the models. Human density entered the model as a second order component as we expected that intermediate densities could increase the conflict level (i.e. intermediate of rural and urban areas). We used mixed models (GLMM) with normal error distribution by entering county as a random effect as each county obtained 4 observations (one for each carnivore species).

#### Wolf zone and sheep farming

To analyse the association between wolf zone and PCI_2_ we used only data from Norway (as Sweden does not have any wolf zone) and estimated PCI_2_ at the municipality level as some counties are split into both zones.

The analyses that include sheep density or sheep loss are only valid for Norway. Around 450 000 sheep are found in Sweden, but they are fenced and highly protected against predation compared to Norway. Consequently sheep depredation is a minor issue in the Swedish carnivore-human conflict. In Norway more than 2 million sheep graze and range freely in the mountains and are much more vulnerable to predation [39]. The numbers we have used for sheep density can be interpreted as sheep available for predation. The analysis on sheep farming was only done on Norwegian data at the county level as sheep numbers are only available at this level.

## Results

### Background Description

There were 2 522 respondents who completed the survey (1 508 in Norway, and 1 014 in Sweden). The response rate was 10% and 15% respectively in the two countries which, according to the collection firm, is a common response rate in telephone surveys (www.norstat.no).

When testing for internal consistency of the data Cronbach’s alpha was higher than 0.80 when using all 16 questions together (α = 0.85), and when separating the questions related to illegal hunting (α = 0.94) and the questions not dealing with illegal hunting (α = 0.82) (Table 1). We checked for multicollinearity between predictor variables at the level of individual respondent. Except for the carnivore densities we found low correlation rates between predictor variables (Spearman’s rank coefficients: -0.47< r_sp_<0.15). The correlation between some of the present carnivore densities was high (e.g. r_sp_ for bears and wolverines = 0.75; and others r_sp_<0.30).

**Figure 1:**
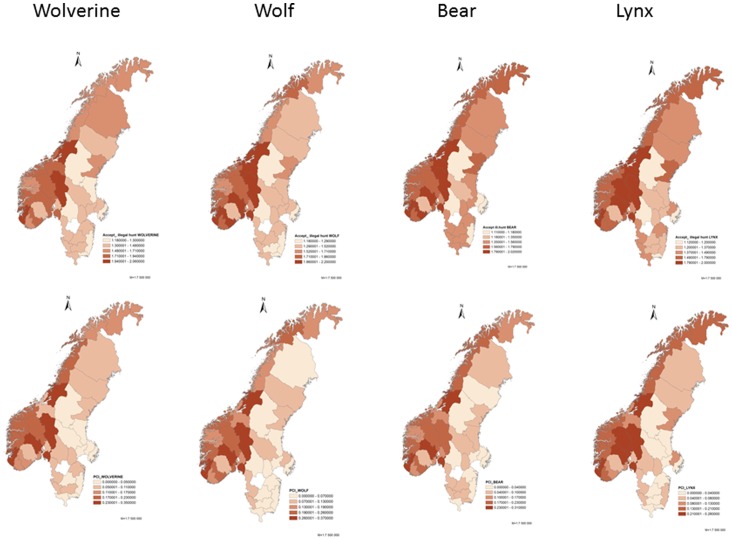
Mapping of mean attitudes towards poaching (upper panel) and mapping of potential conflict index (PCI_2_) in Scandinavia at the county level (lower panel). Dark colours show high acceptance for poaching in a scale from 1 (highly disagree that illegal hunting is acceptable) to 5 (highly agree that illegal hunting is acceptable), and dark colours at the PCI map show where the potential conflict is highest (highest PCI_2_ values). PCI_2_ ranges from 0–1.

There was a high positive correlation between acceptance of poaching and PCI_2_ at both the county and municipality level (0.73< r_sp_<0.91; p<0.001). Hence, the areas with the highest inclination to accept illegal hunting (Fig. 1) are the same areas where we find high PCI_2_ (Fig. 1). This pattern was independent of species and country (Table 2).

**Table 2:** Spearman correlation coefficients (r_sp_ ± SE_r_) between mean acceptance level of poaching and PCI_2_ at the county and municipality level in Norway and in Sweden (all p<0.001).

	County		Municipality	
	Norway	Sweden	Norway	Sweden
	(n = 18)	(n = 19)	(n = 429)	(n = 280)
**Bear**	0.84 (0.14)	0.80 (0.15)	0.80 (0.03)	0.67 (0.04)
**Wolf**	0.88 (0.11)	0.74 (0.16)	0.78 (0.03)	0.65 (0.05)
**Wolverine**	0.84 (0.14)	0.67 (0.18)	0.78 (0.03)	0.59 (0.05)
**Lynx**	0.83 (0.14)	0.73 (0.17)	0.78 (0.03)	0.69 (0.04)

### Acceptance

The acceptance of poaching of the different carnivores was highly correlated (r_sp_>0.86; p<0.001). Hence, if a respondent agreed that it was acceptable to hunt wolves illegally, he or she generally accepted poaching of the other three carnivore species (Table 3). In general, we identified a low level of acceptance of illegal hunting (Table 4).

**Table 3:** Spearman correlation coefficients (r_sp_ ± SE_r_) between acceptance to poach the different carnivore species at the level of the individual respondent (all p<0.001).

	Norway			Sweden		
	(n = 1507)			(n = 1370)		
	Bear	Wolf	Wolverine	Bear	Wolf	Wolverine
**Wolf**	0.84 (0.01)	–		0.80 (0.02)	–	
**Wolverine**	0.86 (0.01)	0.87 (0.01)	–	0.77 (0.02)	0.83 (0.02)	–
**Lynx**	0.84 (0.01)	0.87 (0.01)	0.91 (0.01)	0.77 (0.02)	0.86 (0.01)	0.85 (0.01)

**Table 4:** Mean (95% confidence interval) percentage of respondents who agreed or highly agreed that poaching was acceptable (binomial distribution), and mean (95% confidence interval) potential conflict index (PCI_2_) at county level for each carnivore species.

	Accepted or highly accepted poaching (%)		PCI_2_	
	Norway	Sweden	Norway	Sweden
Bear	13.3 (11.7, 14.9)	3.4 (2.8, 4.1)	0.19 (0.16, 0.21)	0.04 (0.02, 0.07)
Wolf	15.7 (14.1, 17.1)	4.1 (3.4, 5.0)	0.21 (0.19, 0.23)	0.07 (0.04, 0.09)
Wolverine	13.8 (12.3, 15.5)	3.5 (3.0, 4.3)	0.19 (0.17, 0.21)	0.04 (0.02, 0.07)
Lynx	12.7 (11.3, 14.4)	3.3 (2.7, 4.0)	0.18 (0.16, 0.20)	0.04 (0.01, 0.06)

The main analysis of acceptance of illegal hunting was based on a binomial GLMM at the individual level, in order to include the individual characteristics known to affect attitudes towards carnivores as covariates, i.e. sex, age and education level. All three covariates were significant and were included in the model (Table 5). The most parsimonious model for acceptance of illegal hunting at the individual level only included country in addition to the individual characteristics (Table 5). The model showed that respondents in Norway were 4.2 times (95% confidence limit = 3.10, 5.76) more likely to accept poaching than those in Sweden Table 4). There was a slightly higher acceptance of poaching wolves than of the other species (χ^2^ = 7.66, d.f. = 3, p = 0.05) and a positive correlation between acceptance of poaching and a tradition of big game hunting, though this was not significant (logit slope = 0.06± S.E. = 0.03, p = 0.06). Neither present carnivore densities, nor historic carnivore densities were associated with acceptance of poaching (all p>0.1).

**Table 5:** The model of the effect of respondent’s characteristics on acceptance of illegal hunting at the individual level presented with estimates from the logit link function and binomial error.

	Level	Estimate ± SE	?^2^	d.f.	p
Sex	Female	−0.18±0.13	4.57	1	<0.001
	Male	0			
Age		0.016±0.004	26.69	1	<0.001
Education level	Secondary school	0	113.39	3	
	High School	0.41±0.15			<0.001
	University, undergraduate	0.79±0.19			<0.001
	University, graduate	1.36±0.23			<0.001
Country	Norway	0			
	Sweden	1.35±0.15	87.99	1	<0.001

Wolf zone, which is only relevant in Norway, did not improve the statistical models significantly (χ^2^ = 0.05, d.f. = 1, p = 0.82).

**Figure 2:**
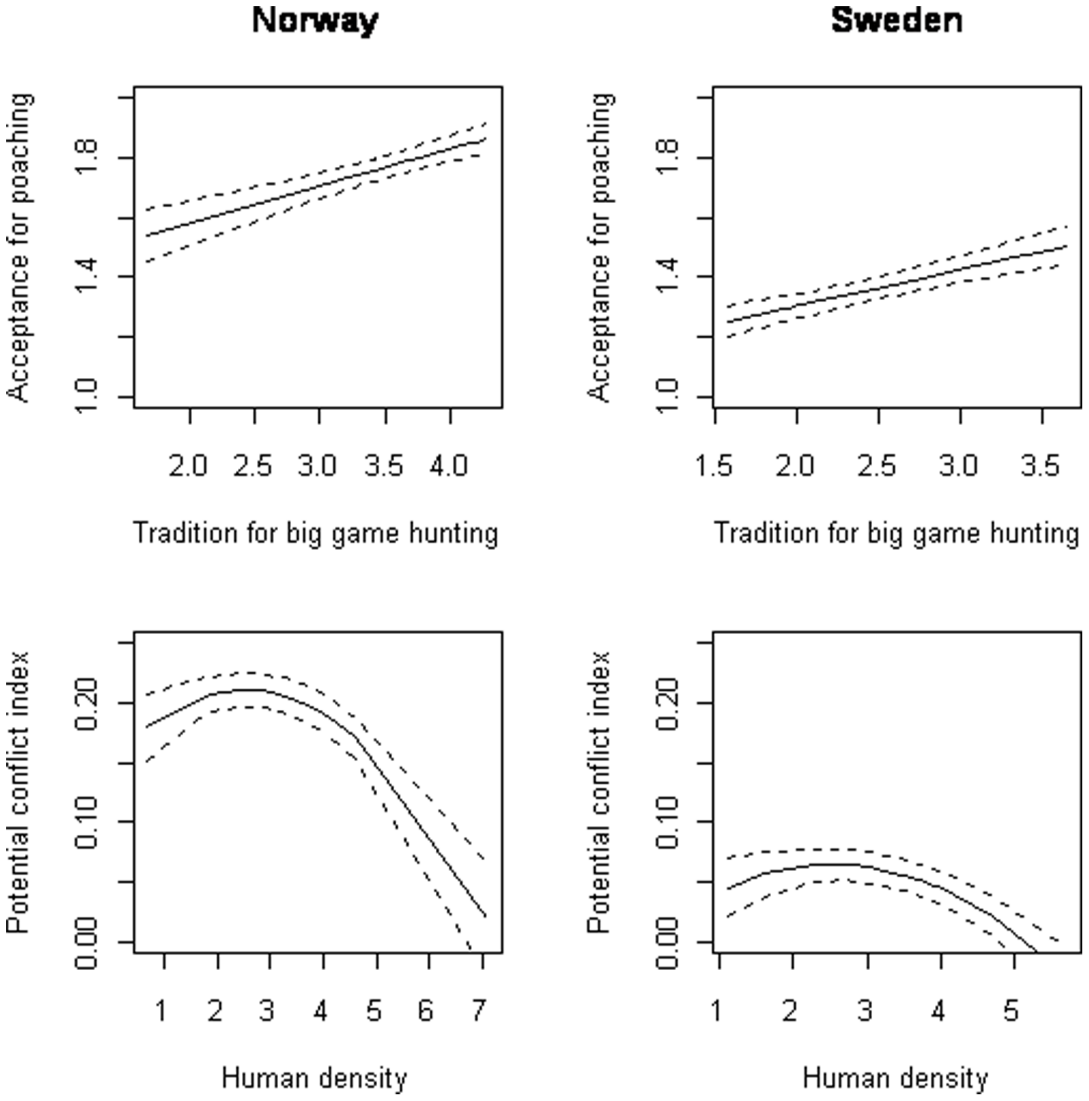
The correlation between acceptance for poaching and traditions for big game hunting (upper panel), and between the potential conflict index (PCI_2_) and human density (log transformed; lower panel) at county level.

As carnivore densities are estimated at the county level we also made a model with average acceptance at each county as response, but without the individual characteristics. The final selected model showed higher acceptance of illegal hunting in Norway than in Sweden (F_1,115_ = 63.98, p<0.001), and a positive correlation between acceptance of illegal hunting and the prevalence of big game hunting (F_1,115_ = 32.49, p<0.001; Fig. 2).

### Conflicts

The selected model showed that the conflict between people (PCI_2_) was higher in Norway than in Sweden (F_1,115_ = 199.10, p<0.001; Table 4) and particularly high in counties with intermediate human densities (F_1,115_ = 9.25, p = 0.004; Fig. 2). Present and historic carnivore densities did not affect the PCI_2_ at county level (all p>0.16).

To test for the effect of wolf zonation we split the data into municipalities, but found no effect of wolf zone on PCI_2_ (all p>0.45).

### Acceptance and Conflicts Related to Sheep Farming

**Figure 3:**
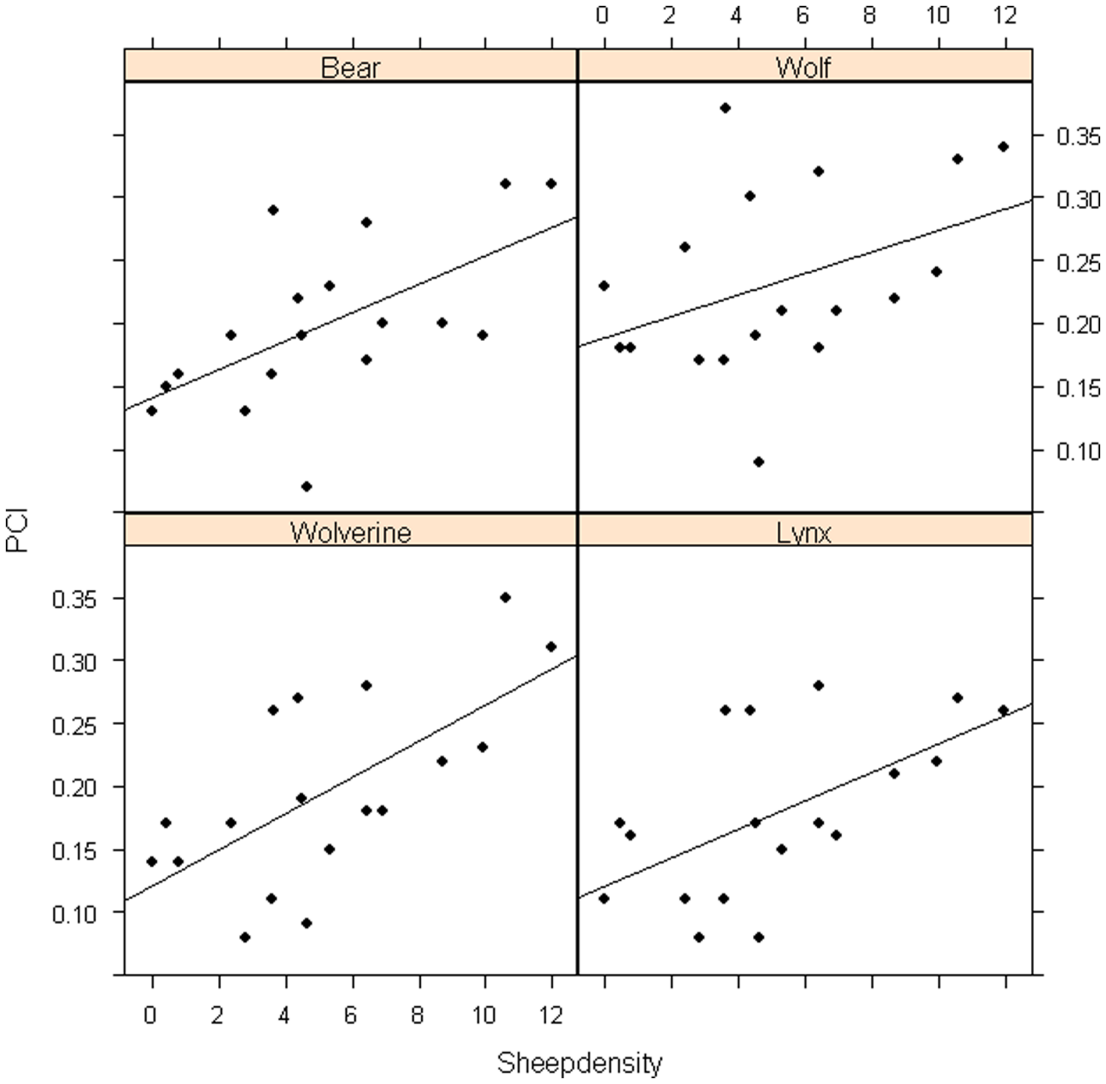
The correlation between potential conflict level (PCI_2_) and free ranging sheep density (sheep pr. km^2^ at county level; log transformed). PCI_2_ estimated for each of the four carnivore species (PCI 6 Bear, PCI 7 Wolf, PCI 8 Wolverine, PCI 9 Lynx).

We only have data of sheep density and sheep loss at the county level in Norway. In these models the acceptance level of poaching was only associated with big game hunting traditions (slope = 0.21, t = 3.3, d.f. = 1, p = 0.004), while PCI_2_ increased with increasing sheep density (slope = 0.064, t = 3.53, d.f. = 1, p = 0.03). We found the same association between PCI_2_ and sheep density regardless of carnivore species (Fig. 3). Carnivore density did not correlate significantly with either acceptance of illegal hunting or PCI_2_ (all p>0.22).

## Discussion

Our findings show that large carnivore conflict in Scandinavia is not driven by the presence of carnivores, the presence of wolf zones, or the loss of sheep depredated by carnivores. Rather, the conflict associated with large carnivores is linked to rural cultural values such as sheep farming and a tradition of big game hunting. People living in rural areas with big game hunting and sheep farming are more likely to accept illegal hunting compared to people living in areas with less rural traditions. Moreover, Norwegians showed a four times higher inclination towards accepting poaching than Swedes. This contrasts with earlier studies showing that attitudes toward wildlife species become increasingly negative when people are directly affected, e.g. when carnivores prey on domestic animals, become a threat to peoples’ safety, or establish themselves close to human settlement [26], [40], [41]. This is often referred to as the NIMBY (Not In My Back Yard) effect – i.e. carnivores might be acceptable until they get too close [42]. If the NIMBY effect was present in our study, we would have expected Swedes to be more inclined to accept poaching than Norwegians since carnivore densities in Sweden are significantly higher than in Norway [14].

Our results showed no differences in attitude toward poaching within and outside the wolf zoning area. This is supported by our finding that carnivore abundance did not affect the attitude toward poaching and that those who accept poaching do so independent of carnivore species. Therefore, zonation for one species may not be the best solution to reduce acceptance of illegal hunting.

Part of the explanation for the variation between Norway and Sweden might be how people identify with rural cultures. While 63% of the Norwegian respondents in our survey report they are living in a municipality with strong big game hunting traditions, this was the case for only 23% of the Swedish respondents. Although Norway and Sweden share many social, cultural and geographical characteristics, one major difference is the Norwegian district policy by which the government subsidises rural settlements and economic development throughout the country, including agriculture and livestock husbandry [43]. This action helps to maintain the cultural landscape shaped by grazing and small scale farming despite the fact that the national sheep industry is not economically sustainable the way it is structured today [44]. After decades of bounties and eradication of large carnivore populations in Norway [41], [45], [46], more than 2 million sheep currently graze freely on pastures, forest or mountain ranges without any protection from carnivores [47], [48]. When the carnivores returned to Norway due to conservation efforts in the 1960s and 1970s [41], the rural practice of free-ranging sheep in Norway did not adapt to this changing situation. Switzerland, a country economically and politically comparable to Norway and Sweden shows a similar pattern in their human – carnivore conflict. According to Breitenmoser (1998) the return of large predators will not be possible in Switzerland without changing the system of sheep husbandry [49].

Our findings also showed that people in areas with big game hunting traditions were more accepting of poaching. Lagendijk and Gusset (2008) points out the importance of taking cultural values and cultural differences into considerations when dealing with human – carnivore conflicts [50]. They found higher tolerance for lions in a rural society which had always been positive to predators compared to similar societies where people had a tradition of being more hostile in their attitude toward lions and were known for persecuting large carnivores [50]. Therefore, sheep abundance and big game hunting might be important factors in how people identify with rural cultures and values. It can be argued that poaching in Scandinavia is to a large extent motivated by the rural-urban divide where rural residents show opposition to urbanization, urban values, central authorities and regulations [11], [12]. Additionally, big game hunters tend to be more negative toward large carnivores and more accepting of poaching because they are competing with the carnivores for the same prey e.g. moose (*Alces alces*) or deer (*Cervus elaphus*) and high predation rates from carnivores usually impact the hunting quotas in a negative way [51]. If wolves establish a territory in a moose hunting area, the moose quotas might be reduced to adjust for wolf predation [26].

According to the Potential Conflict Index, we found a greater potential for conflict among people in areas where free-ranging sheep are common; however, sheep depredation did not affect the potential conflict level. Counties with intermediate human density showed a greater discrepancy in people’s opinions about illegal hunting compared to areas with low or high densities. We interpret these areas of intermediate population density to be mixed rural-urban areas such as small towns, rural areas with increased urbanisation, or areas where rural values and old traditions live side-by-side with more modern urban life styles. Intermediate human density areas might represent counties where, for instance, a high proportion of younger people move back from big cities after completing higher education. The younger, more educated generation thereby represent a potential contrast in how nature is viewed relative to the old traditions and farming values. Societies or counties with low human densities might be rural and more homogeneous areas, and, therefore, more coherent in their own opinion.

### Conclusion

Previous research on human – wildlife conflicts have largely ignored or only superficially treated the spatial dimensions of attitudes, and focused instead on how demographic parameters such as age, sex and education affect attitudes [36], [52], [53]. To better understand the conflicts and to guide the choice of potential management actions, there is a need for more information on whether people’s attitudes vary across larger geographic regions, how people react to being part of a zoning area, and to what extent attitudes are formed in relation to management interventions like zoning schemes. We revealed that areas with people that show higher acceptance of illegal hunting are areas with high potential conflict. The present study suggests that negative attitudes toward carnivores relate primarily to rural values, cultures and identity such as sheep husbandry and big game hunting. Areas where rural values conflict with more urban values experience a higher conflict level and people living in rural areas are more prone to accept poaching, whether or not there are local carnivores. Any establishment of carnivore populations in such areas will meet with substantial resistance [21], [36], [54]–[56].
